# Publisher’s Note: Shuttle-box systems for studying preferred environmental ranges by aquatic animals

**DOI:** 10.1093/conphys/coab051

**Published:** 2021-06-29

**Authors:** Emil A F Christensen, Lars E J Andersen, Heiðrikur Bergsson, John F Steffensen, Shaun S Killen

*Conservation Physiology*, Volume 9(1): coab028, doi: 10.1093/conphys/coab028

An earlier version of this manuscript, which had not been approved by the authors, was inadvertently published. As a result, the manuscript contained several grammatical and formatting errors. The following references were also included in error:

- Elsner RA, Shrimpton JM (2019) Behavioural changes during the parr–smolt transformation in Coho salmon *Oncorhynchus kisutch*: is it better to be cool? *J Fish Biol* 95: 793–801.
- Christensen EAF, Grosell M, Steffensen JF (2019) Maximum salinity tolerance and osmoregulatory capabilities of European perch *Perca fluviatilis* populations originating from different salinity habitats. *Conserv Physiol* 7: 1–5.
- Fredricks K, Tix J, Smerud J, Cupp A (2020) Laboratory trials to evaluate carbon dioxide as a potential behavioral control method for invasive red swamp (*Procambarus clarkii*) and rusty crayfish (*Faxonius rusticus*). *MBI* 11: 259–278.
- Crawshaw LI, Hammel HT (1971) Behavioral thermoregulation in two species of Antarctic fish. *Life Sci* 10: 1009–1020.
- Stich DS, Zydlewski GB, Zydlewski JD (2016) Physiological preparedness and performance of Atlantic salmon *Salmo salar* smolts in relation to behavioural salinity preferences and thresholds. *J Fish Biol* 88: 595–617.
- Reynolds WW, Casterlin ME (1978a) Thermoregulatory behavior in smooth dogfish shark, *Mustelus canis*. *Fed Proc* 37: 427–427.
- Reynolds WW, Casterlin ME (1978b) Behavioral thermoregulation by Ammocoete larvae of the sea lamprey (*Petromyzon marinus*) in an electronic shuttlebox. *Hydrobiologia* 61: 145–147.
- Reynolds WW, Casterlin ME (1979) Thermoregulatory behaviour of the primitive arthropod *Limulus polyphemus* in an electronic shuttlebox. *J Therm Biol* 4: 165–166.
- Schurmann H, Steffensen JF (1997) Effects of temperature, hypoxia and activity on the metabolism of juvenile Atlantic cod. *J Fish Biol* 50: 1166–1180.

The final version of this manuscript has now replaced this earlier version, and so these errors are no longer present. The publisher apologizes for this mistake and regrets any confusion caused.

